# Effects of lipopolysaccharide-induced inflammation on hypoxia and inflammatory gene expression pathways of the rat testis

**DOI:** 10.1186/s12610-018-0079-x

**Published:** 2018-11-16

**Authors:** Michael A. Palladino, Genevieve A. Fasano, Dharm Patel, Christine Dugan, Marie London

**Affiliations:** Monmouth University, 400 Cedar Avenue, West Long Branch, NJ 07764 USA

**Keywords:** Hypoxia, Orchitis, Pathogens, Lipopolysaccharide, Inflammation, Hypoxie, Orchite, Pathogènes, Lypopolysaccharides, Inflammation

## Abstract

**Background:**

Bacterial infection and inflammation of the testis impairs fertility, yet an understanding of inflammatory responses of the testis is incomplete. We are interested in identifying gene pathways involved in the detection and clearance of infectious microbes in the male reproductive tract. In previous studies in our lab focused on hypoxia-responsive genes of the testis, preliminary experiments suggested that genes classically categorized as hypoxia genes are also activated during antimicrobial responses. The purpose of this study was to identify hypoxia and inflammatory gene pathways that contribute to antimicrobial protection of the testis and to consider possible cross-talk and interactions between these pathways. Inflammation was induced in Sprague-Dawley rats using *P. aeruginosa* or *E. coli* lipopolysaccharide (LPS). Levels of hypoxia-inducible factor-1 (HIF-1α) protein and nuclear factor kappa B (NF-κB) were measured, and hypoxia and inflammatory gene expression patterns in testis were analyzed by gene expression profiling using real-time quantitative PCR arrays.

**Results:**

In LPS-treated rats, HIF-1α protein increased with no change in *Hif-1α* mRNA. Western Blot analysis also demonstrated no change in NF-κB and inhibitory NFKB alpha (IκBα) protein levels following LPS treatment. Five hypoxia pathway genes (*Angptl4, Egr1, Ier3, Pai1,* and *Glut1)*, and 11 inflammatory pathway genes (*Ccl12, Cc13, Cd14, Cxcl10, Icam1, Il10, Il1b, Il6, Nfkbia, Tlr2, Tnf)* up-regulated after 3 h of inflammation. *Angptl4, Ccl12, Cc13, Cd14, Egr1, Nfkbia, Tlr2,* and *Tnf* remained elevated at 6 h. Six genes were up-regulated at 6 h only (*Bhlhe40*, *C3, Jak2, Nlrp3, Slc11a1, Tlr1)*. One gene (*Tlr5*) was down-regulated after 3 h and no genes at 6 h. Electrophoretic mobility shift assay results suggest a decrease in NF-κB binding activity following LPS treatment.

**Conclusions:**

Testicular HIF-1α is up-regulated following LPS-induced inflammation. In contrast to other tissues, in which HIF-1α is up-regulated through transcriptional activation via NF-κB, we conclude that HIF-1α in the testis is not up-regulated through an increase in *Hif-1α* mRNA or through NF-κB-dependent mechanisms. Hypoxia pathway genes and genes involved in Toll-like receptor (TLR) and cytokine-mediated signaling comprise major functional categories of up-regulated genes, demonstrating that both hypoxia and classic inflammatory pathways are involved in inflammatory responses of the testis.

**Electronic supplementary material:**

The online version of this article (10.1186/s12610-018-0079-x) contains supplementary material, which is available to authorized users.

## Résumé

**Contexte:**

L’infection et l’inflammation bactériennes du testicule altèrent la fertilité; cependant la compréhension des réponses inflammatoires du testicule est. encore incomplète. Nous nous sommes intéressés à l’identification des voies des gènes impliqués dans la détection et l’élimination des microbes infectieux dans l’appareil reproductif masculin. Dans de précédentes études menées dans notre laboratoire, et centrées sur des gènes sensibles à l’hypoxie, les expérimentations préliminaires suggéraient que les gènes classiquement catégorisés comme gènes de l’hypoxie étaient aussi activés au cours des réponses antimicrobiennes. Le but de la présente étude était d’identifier les voies des gènes qui contribuaient à la protection antimicrobienne du testicule et d’examiner de potentiels intermodulations et interactions entre ces voies.

L’inflammation a été induite chez des rats Sprague-Dawley en utilisant des lypopolysaccharides (LPS) de *P. aeruginosa*et d’*E. coli*. Les taux de protéine du facteur-1 inductible par l’hypoxie (HIF1- α) et du facteur nucléaire kappa B (NF- _k_B) ont été mesurés; les profils d’expression des gènes de l’hypoxie et de l’inflammation dans le testicule ont été analysés par profilage de l’expression génique par PCR quantitative en temps réel.

**Résultats:**

Chez les rats traités par LPS, la protéine HIF-1 α a augmenté sans modification de *Hif-1α*mRNA. L’analyse par Western Blot a aussi montré l’absence de modifications des taux de NF-_k_B et de la protéine inhibitrice NFKB alpha (I_k_B α) après traitement. Cinq gènes de la voie hypoxie (*Angptl4, Egr1, Ier3, Pai1,*et *Glut1*), et 11 gènes de la voie inflammatoire (*Ccl12, Cc13, Cd14, Cxcl10, Icam1, Il10, Il1b, Il6, Egr1, Nfkbia, Tlr2*, et *Tnf*) ont été régulés à la hausse après 3 heures d’inflammation. *Angptl4, Ccl12, Cc13, Cd14, Egr1, Nfkbia, Tlr2*, et *Tnf*sont restés élevés à 6 heures. Six gènes n’ont ont été régulés à la hausse qu’à 6 heures (*Bhlhe40, C3, Jak2, Nlrp3, Slc11a1, Tlr1*). Un gène (*Tlr5*) a été régulé à la baisse après 3 heures et aucun gène à 6 heures. Les résultats du test de décalage de la mobilité électrophorétique suggèrent une baisse de l’activité de liaison de NF- _k_B après traitement par LPS.

**Conclusions:**

HIF-1α testiculaire est. régulé à la hausse après inflammation induite par LPS. Contrairement à d’autres tissus, dans lesquels HIF-1α est. régulé à la hausse par activation transcriptionnelle via NF- _k_B, nous concluons que HIF-1α dans le testicule n’est. pas régulé à la hausse par une augmentation de *Hif-1 α*mRNA ou par des mécanismes NF-_k_B-dépendants. Les gènes de la voie hypoxie et les gènes impliqués dans le récepteur Toll-like (TLR) et dans la signalisation médiée par les cytokines comprennent des catégories fonctionnelles majeures de gènes régulés à la hausse, ce qui démontre qu’à la fois les voies de l’hypoxie et les voies classiques de l’inflammation sont impliquées dans les réponses inflammatoires du testicule.

## Background

Infection and resulting inflammation of male reproductive organs impairs fertility by decreasing sperm mobility through the tract and reducing androgen production among other effects on spermatozoa and on the testis and epididymis [1–3]. Roughly half of all infertility cases are caused by male infertility and over 30% of male infertility is idiopathic, yet the relevance of infection and inflammation in male infertility, although widely studied clinically, is still subject to debate [4, 5]. Research to better understand the effects of pathogens on the male reproductive tract and cellular and molecular mechanisms involved in the detection and response to infection of male reproductive organs is essential.

In vivo and in vitro models involving the administration of lipopolysaccharide (LPS), a major pathogenic component of the cell wall of Gram negative bacteria, is a common experimental approach to evaluate inflammatory responses [6, 7]. Pathogen-specific recognition sensors, such as Toll-like receptors (TLR), have been identified in the testis and implicated in the physiology of Leydig, Sertoli and spermatogenic cells [8, 9]. It is well-known that across species a number of antimicrobial peptides are produced by the testis including *Spag11*, members of the defensin family, *Eppin* and others [8, 10–17].

Effects of uropathogenic bacteria and LPS in the testis include inhibition of steroidogenesis and reduced androgen production [18, 19], decreased intracellular cAMP and reduced sperm motility [20], induction of proinflammatory cytokines and activation of antimicrobial pathways [10], and epigenetic regulation of antimicrobial gene expression [21]. However, relatively little is known about interactions between regulatory pathways and mechanisms involved in the response to inflammation of the testis.

In other tissues, cross-talk between hypoxia regulatory pathways and classic inflammatory pathways has been demonstrated [22, 23]. This led us to hypothesize that both pathways contribute significantly to inflammatory responses of the testis. We were intrigued by this hypothesis in part because of our prior work on testis hypoxia and the transcription factor hypoxia-inducible factor-1 (HIF-1). Recognized as the master regulator of hypoxia, HIF-1 is known to activate transcription of over 100 genes crucial for cellular responses to hypoxia [24–26]. Increasingly, HIF-1 activation has been implicated in a range of inflammatory responses [27]. For example, HIF-1 is induced by proinflammatory cytokines such as tumor necrosis factor alpha (TNF-α) and interleukin beta (IL-1ß) [28], and by LPS under normoxic conditions [29].

HIF-1α is expressed by Leydig cells in the normoxic adult rat testis and is unregulated by hypoxia [30]. We have also demonstrated that myeloid leukemia cell differentiation 1 (*Mcl-1*) is a target gene for testicular HIF-1 with potentially important roles in antiapoptotic protection of Leydig cells [31]. Understanding mechanisms of HIF-1 activation under normoxic, hypoxic, and other physiological conditions, such as inflammation, in the testis is of significant interest to investigators interested in the roles of HIF-1 on Leydig cell physiology.

The present study was initiated to (1) investigate whether HIF-1 is affected by LPS-induced inflammation of the testis as an initial step to determine if co-activation of hypoxia and inflammatory pathways occurs in the testis, (2) determine if other hypoxia pathway genes are activated by inflammation, and (3) to explore potential cross-talk between expression of hypoxia and inflammatory pathway genes.

## Methods

### Animals

Adult male, retired-breeder Sprague-Dawley rats (475–750 g, 9–12 months, Charles River Laboratories (Stone Ridge, NY) were housed individually on a 12:12-h light/dark cycle and controlled temperature with free access to food and water.

### Administration of Lipopolysaccharides

*P. aeruginosa* LPS (Type 10, L7018; Sigma-Aldrich, St. Louis, Missouri) and *E. coli* 055:B5 LPS (L2880; Sigma-Aldrich) were selected because both are from known pathogens of the urogenital tract and cause tissue-specific inflammatory responses. A dosage of 5.0 mg/kg body weight was chosen to maximize activation of inflammatory responses. Others have clearly demonstrated LPS doses that generate inflammatory responses in vivo (1–5 mg/kg body weight) and in vitro (0.1–1.0 mg/ml), models [10, 16, 18, 19, 32]. LPS was reconstituted in sterile 1× phosphate buffered saline (PBS; 10 mM sodium phosphate, 150 mM sodium chloride, pH 7.8) and administered via intraperitoneal injection. Sham controls were injected with sterile PBS. After 1, 3, 6, and 12 h of treatment (*n* = 5–7/time point), animals were euthanized via CO_2_ gas. Testes and control tissues were excised then frozen in liquid nitrogen and stored at − 80 °C.

### Serum testosterone assays

Blood was collected via cardiac puncture at the time of sacrifice, clotted at room temperature for 30 min, centrifuged at 13,000 rpm for 10 min at 4 °C then sera was removed and stored at − 80 °C prior to testosterone measurements. Testosterone radioimmunoassays were carried out using a Packard 1900TR Liquid Scintillation Analyzer (Canberra, Meriden, CT). Internal controls included a no testosterone control and a positive control sample of 100 ng/ml testosterone.

### Protein extraction

Frozen tissue samples were homogenized on ice in three volumes of lysis buffer (10 mM Tris-HCL [pH 7.5], 1.5 mM MgCl_2_, 1 mM dithiothreitol, 1 mM Na_3_VO_4_ containing protease inhibitor cocktail; P8340; Sigma-Aldrich). The homogenate was maintained on ice for 10 min then centrifuged for 5 min at 3500 rpm at 4 °C. Supernatant was removed for cytoplasmic proteins. Nuclei were resuspended in three volumes of 0.42 M KCl, 20 mM Tris-HCL (pH 7.5), 1.5 mM MgCl_2_, 20% glycerol, mixed for 30 min at 150 rpm at 4 °C, and centrifuged at 13,500 rpm for 30 min at 4 °C to isolate nuclear proteins. Protein extracts were stored at − 80 °C and protein concentrations determined by the Bradford assay (Bio-Rad, Hercules, CA).

### Immunoblot analysis

Proteins were separated by denaturing sodium dodecyl sulfate polyacrylamide gel electrophoresis (SDS-PAGE) through 7.5% PAGEr® Gold Precast Gels (Lonza, Rockland, MD) in 1× Tris-glycine SDS buffer (0.25 mM Tris, 192 mM glycine, and 0.1% (*w*/*v*) SDS [pH 8.3]). COS-7 simian virus 40-transformed kidney cell nuclear extract (Active Motif, Carlsbad, CA) was included as a positive control for detecting HIF-1α. RAW 264.7 cell extract (Santa Cruz Biotechnology, Santa Cruz, CA) was included as positive control for NF-κB and IκBα. Proteins were electroblotted onto Trans-Blot nitrocellulose (Bio-Rad), blocked with 1× Western wash (50 mM Tris, 30 mM NaCl, 0.001% Tween 20 [pH 7.6]) containing 5% nonfat dry milk (NFDM) for 30 min at room temperature, then incubated in primary antibody overnight at 4 °C in 1× Western wash, 5% NFDM.

Primary antibody dilutions were as follows: 0.5 μg/mL HIF-1α mouse monoclonal antibody (AF1935; R&D Systems, Minneapolis, MN), 0.5 μg/mL NF-κB rabbit polyclonal antibody (ADI-KAP-TF112, Enzo Life Sciences, Farmingdale, NY), 0.5 μg/mL IκB rabbit polyclonal antibody (9242; Cell Signaling, Danvers, MA). Actin was detected as a loading control for protein quantification using a 1:2000 dilution of rabbit polyclonal antibody (A2066, Sigma-Aldrich). Blots were incubated in 1× Western wash, 5% NFDM containing appropriate horseradish peroxidase (HRP)-conjugated secondary antibodies (1:20,000 dilution), washed in 1× Western wash, developed by enhanced chemiluminescence using either SuperSignal® West Pico substrate or SuperSignal® West Femto substrate (Pierce, Rockford, IL), and analyzed with a ChemiDoc™ XRS+ Molecular Imager with Image Lab™ Software (Bio-Rad).

### Gene expression profile analysis by real-time quantitative PCR (qPCR)

Total RNA was isolated from frozen tissue using TRIzol reagent (Invitrogen, Grand Island, NY). Gene expression profiles for hypoxia pathway genes and inflammatory pathway genes were analyzed using Rat Hypoxia Signaling Pathway and Rat Innate and Adaptive Immune Response Pathway RT^2^ Profiler™ PCR Arrays (Qiagen, Frederick, MD). One microgram of genomic-DNA-free total RNA was reverse transcribed using the RT^2^ First Strand cDNA Kit (330,401, Qiagen). Real-time qPCR assays were carried out with SYBR Green dye and amplified for 40 cycles in a Stratagene Mx3500P® thermocycler. Each array contained 84 genes involved in the hypoxia or innate and adaptive immune response pathways. Listings of all genes queried and accession numbers are available from the Qiagen website.

Positive controls and housekeeping genes were included to normalize for differences in sample loading, genomic DNA contamination, and amplification efficiency. PCR controls included no RT and no template negative controls. Relative quantification of all target genes was calculated by first normalizing comparative cycle threshold (C_t_) values of target genes to ß-actin. Normalized values were used to calculate target gene expression in treatment groups compared to shams. C_t_ values of controls were ~ 8–10 cycles beyond RT test samples indicating that no contaminating DNA was present. Genes that displayed an average (*n* = 4–7) minimum of three-fold change (up- or down-regulation) were considered statistically significant (*p* < 0.05) by analysis of variance (ANOVA).

### In silico analysis

Computer-based (in silico*)* methods were utilized for the analysis of candidate genes involved in inflammatory responses of the testis. Methods included literature searches using PubMed (http://www.ncbi.nlm.nih.gov/pubmed/) and bioinformatics databases such as UniProt (https://www.uniprot.org) for gene expression and protein distribution data. These electronic resources were used to determine if there was existing data available about these genes, such as expression and regulation data, cell-type-specific expression in the testis, and protein expression data from other researchers. Information about each gene of interest was gathered to propose pathway maps. The Database for Annotation, Visualization, and Integrated Discovery (DAVID) Bioinformatics Resources 6.7 available through the National Institute of Allergy and Infectious Diseases (NIAID) was utilized for functional annotation and pathway map analysis (https://david.ncifcrf.gov).

### Electrophoretic mobility shift assays

Electrophoretic Mobility Shift Assays (EMSA) were performed using the Thermo Scientific LightShift® Chemiluminescent EMSA kit according to manufacturer’s instructions (Thermo Scientific, Rockford, IL). Terminal deoxynucleotidyl transferase (TdT) was used to catalyze biotin labeling of oligonucleotides (Biotin 3′ End DNA Labeling Kit, Pierce). Binding reactions were carried out in 20 μl volumes with protein extracts incubated with 10× binding buffer, 1 μg/μl poly (dI•dC), 50% glycerol, 1% NP-40, 1 M KCl, 100 mM MgCl_2_, and 200 mM EDTA and biotin-labeled oligonucleotide for 20 min at room temperature. Epstein-Barr Nuclear Antigen (EBNA) and biotin-labeled double-stranded target DNA were included as positive controls. Unlabeled EBNA oligonucleotides were used in negative control and competition experiments. NF-κB double-stranded oligonucleotide with a consensus binding site for NF-κB /c-Rel homodimeric and heterodimeric complexes (5’-AGTTGAGGGGACTTTCCCAGGC -3′; sc-2505, Santa Cruz Biotechnology) which is similar to the sequence present in the upstream region of the HIF-1 gene and mutant oligonucleotides (sc-2511) with a C-G substitution in the consensus site were used for EMSA.

EMSA reactions were separated through 6% polyacrylamide TBE PAGEr® Gold gels (Lonza) electrophoresed in 0.5× tris-borate-EDTA buffer (AccuGENE® 10× TBE Buffer, Lonza), transferred onto nylon (Biodyne® B, Thermo Scientific) and membranes cross-linked at 120 mJ/cm^2^ for 60 s using a UV-light cross-linker. Blots were blocked, incubated with a 1:300 dilution of stabilized streptavidin-HRP conjugate for 15 min at room temperature, then reacted with luminal/enhancer solution. Digital images were captured with a ChemiDoc™ XRS+ quantitative analysis performed using Image Lab™ Software.

## Results

### Serum testosterone levels decrease following LPS treatment

**Fig. 1:**
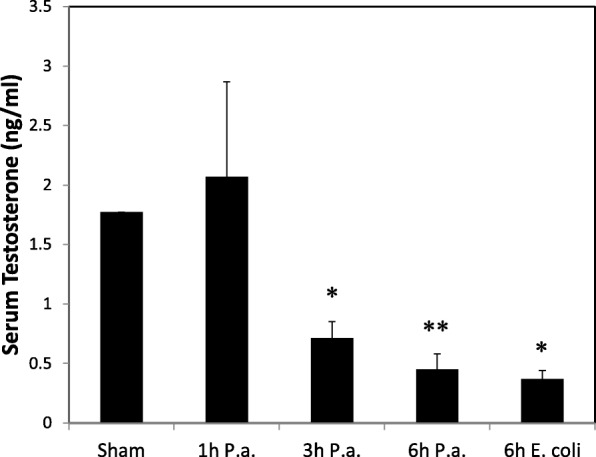
Serum testosterone levels decrease following LPS-induced inflammation. *Indicates statistically significant difference as compared to sham (ANOVA, *P* < 0.05; *n* = 3–5); **Indicates *P* < 0.001. P.a., *Pseudomonas aeruginosa*

A rat model of orchitis, or testicular inflammation, was established via administration of *P. aeruginosa* or *E. coli* LPS at a dosage of 5 mg/kg bw. It is well-established that one effect of LPS on the testis is diminished testosterone output and reductions in plasma testosterone levels [19]. To determine if 1 h, 3 h, and 6 h *P. aeruginosa* and 6 h *E. coli* LPS treatment caused acute inflammation of the testis and the expected reduction in circulating testosterone, serum testosterone levels were measured via radioimmunoassay. No decrease in serum testosterone levels was observed at 1 h *P. aeruginosa* LPS treatment (Fig. 1). Statistically significant decreases in serum testosterone levels were observed at 3 h and 6 h *P. aeruginosa* and 6 h *E. coli* LPS treatment as compared to sham (*P* < 0.05; Fig. 1), demonstrating the expected and well-reported physiological effect of LPS inflammation on the testis under our experimental conditions.

Increased expression of *Il-6* has been reported in the testis following LPS-induced inflammation at both 3 and 9-h time points [21]. As mentioned later in the manuscript, we detected elevated mRNA expression for *Il-6* mRNA in 3 h LPS treatment groups providing further evidence for inflammation of the testis under these experimental conditions (see Table 2).

### HIF-1α protein levels increase following LPS-induced inflammation

**Fig. 2:**
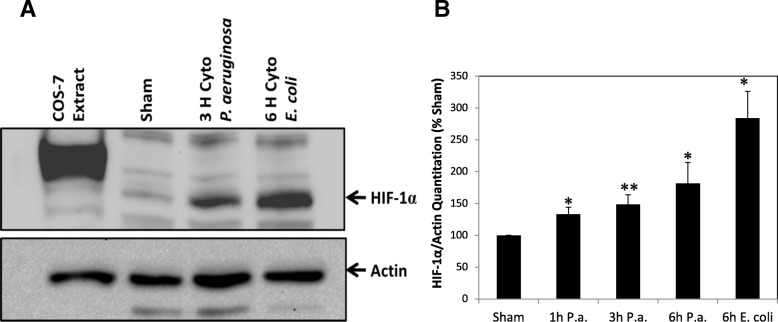
HIF-1α protein levels increase following LPS-induced inflammation in rat testis. **a** Results of HIF-1α immunoblot analysis of testicular cytoplasmic proteins isolated from sham and LPS treated animals. COS-7 cell line protein extract was included as a positive control and ß-actin was included as an internal control. HIF-1α was detected at ~ 120 kDa. Shown is a 6 h sham as an example but time-matched shams (1, 3 and 6 h) were used for all experiments and for quantitation. Representative time points and results are shown in panel (**a**). **b** Histogram shows relative HIF-1α protein levels normalized to ß-actin and as compared to sham for all time points and treatment samples analyzed. *Indicates statistically significant difference as compared to sham (ANOVA, *P* < 0.05; *n* = 4–7). **Indicates *P* < 0.001

To determine if testis inflammation results in the activation of hypoxia pathways through HIF-1, HIF-1α protein levels in testis from sham and treatment groups were detected by Western blot analysis (Fig. 2a). Statistically significant increases in HIF-1α protein were observed at 1 h, 3 h and 6 h *P. aeruginosa* LPS treatment as compared to sham and following 6 h *E. coli* LPS treatment (Fig. 2b). In an initial pilot study to determine time course and LPS dosage, 1 h and 3 h *E. coli* LPS treatment experiment was performed but no apparent changes in HIF-1α protein were detected (data not shown), therefore it appears that *P. aeruginosa* LPS produces a more acute phase response in elevating HIF-1 than *E. coli* LPS*.*

### NF-κB and IκB protein levels following LPS-induced inflammation

**Fig. 3:**
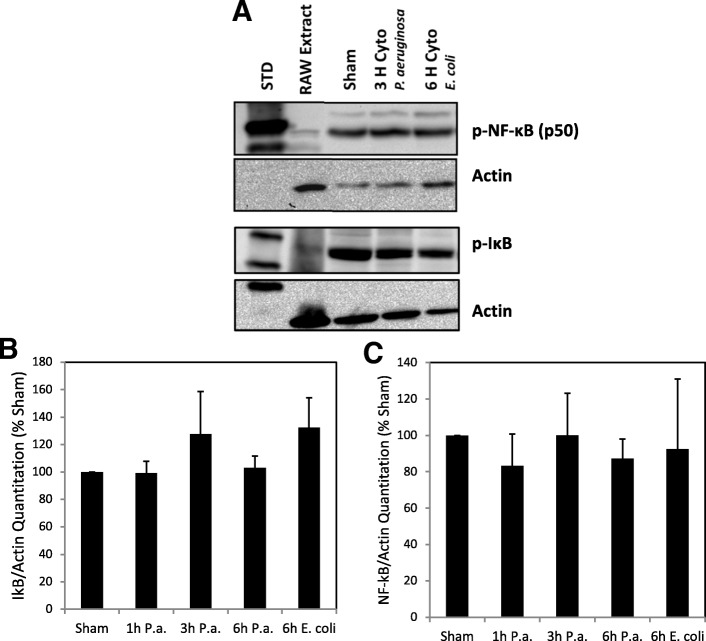
Phospho-NF-κB and IκB protein levels do not change following LPS-induced inflammation in rat testis. **a** Results of phospho-NF-κB and phospho-IκB immunoblot analysis of testicular cytoplasmic proteins isolated from sham and LPS treated animals. RAW cell line protein extract was included as a positive control and ß-actin was included as an internal control. Representative time points and results are shown in panel (**a**). Histograms show relative phospho-IκB (**b**) and phospho-NF-κB (**c**) protein levels normalized to ß-actin and as compared to sham for all time points and treatment samples analyzed. No statistically significant differences in phospho-IκB and phospho-NF-κB protein levels were observed between sham and LPS treated groups (ANOVA, *P* < 0.05; *n* = 3–5). STD, protein molecular weight standards

NF-κB is a major activator of inflammatory pathways and has been shown to transcriptionally regulate HIF-1 under conditions of inflammation in other cell types [33, 34]. In an effort to identify the mechanism(s) responsible for HIF-1α protein accumulation following LPS-induced inflammation of the testis, phospho-NF-κB and phospho-IκB protein levels in testicular cytoplasmic protein extracts were measured via Western blot analysis. No differences in phospho-NF-κB and phospho-IκB protein levels were observed in the testis following LPS-induced inflammation at all time points as compared to sham (*P* < 0.05; Figs. 3a-c).

### NF-κB DNA binding activity decreases following LPS-induced inflammation

**Fig. 4:**
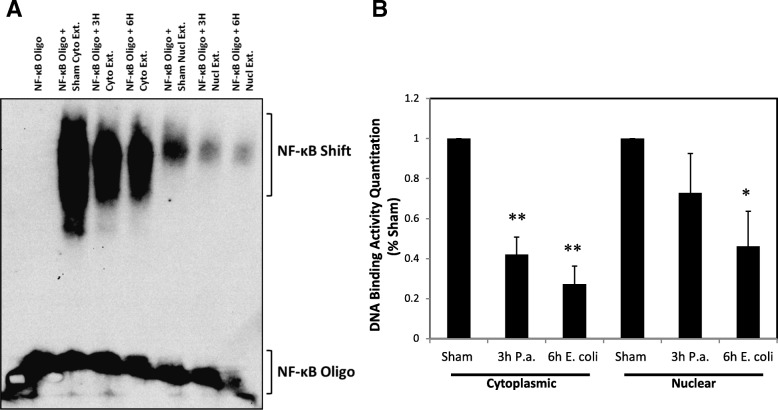
Electrophoretic mobility shift assay (EMSA) for NF-κB binding to the HIF-1α gene. **a** NF-κB binding to a consensus sequence from the HIF-1α gene in both cytoplasmic and nuclear protein extracts decreased following LPS treatment. **b** Relative DNA binding activity as compared to sham. *Indicates statistically significant difference as compared to sham (ANOVA, *n* = 3–5; *P* < 0.05). **Indicates *P <* 0.001

EMSAs were performed to evaluate the DNA binding activity of NF-κB. An oligonucleotide with a consensus binding site for NF-κB homo- and heterodimeric complexes, similar in sequence to a binding site upstream of the HIF-1 gene, was used for EMSA with both testicular cytoplasmic and nuclear protein extracts (Fig. 4a). These time points were chosen to determine if there was any difference in inflammatory response caused by the different treatments. A decrease in binding activity was observed in the cytoplasmic extracts of both 3 h *P. aeruginosa* and 6 h *E. coli* LPS treatment and nuclear extracts of 6 h *E. coli* LPS treatment as compared to sham (*P <* 0.05; Fig. 4b).

### Gene expression profiling of hypoxia pathway genes

That HIF-1 increased following LPS-induced inflammation suggests roles for hypoxia regulated genes in inflammatory responses of the testis. Gene expression profiling arrays were used to identify hypoxia pathway genes that are regulated following LPS administration. Also, to determine if regulation of HIF-1α protein accumulation following LPS-induced inflammation of the testis occurs at the transcriptional level, *Hif-1α* mRNA levels were measured in these arrays. No statistical significant difference in *Hif-1α* mRNA levels was observed at any time point following LPS-induced inflammation in the testis by the arrays or by independent RT-PCR assays for *Hif-1α* (data not shown), suggesting that HIF-1 protein up-regulation is occurring at the post-transcriptional level. Stabilization and degradation of HIF-1 protein is very well characterized as the primary mechanism controlling HIF-1 protein levels [26].

Pathway array results demonstrated that 5 genes in the hypoxia pathway, *Angptl4, Egr1, Ier3, Pai1,* and *Slc2a1 (Glut1)*, were up-regulated after 3 h of LPS-induced inflammation and expression and showed more than a three-fold change (*p* < 0.05; Table 1). Following 6 h of LPS-induced inflammation, 3 of these genes (*Angptl4, Bhlhe40,* and *Egr1),* showed statistically significant up-regulation (Table 1). No genes were down-regulated following LPS administration. In silico analysis of LPS-stimulated genes indicates that these transcripts are predominantly expressed in supporting cells of the testis (Sertoli, myoid, and Leydig cells) and not in developing germ cells. *Egr1, Fos, Ier3,* and *Pai1* are known target genes for the transcription factor NF-κB. None of the up-regulated genes are known HIF-1 targets.

**Table 1:** Up-regulation of hypoxia pathway genes after 3 and 6 h of LPS-induced inflammation

Gene Name Unigene, GenBank	Fold Change	
	*3 h (n = 7)*	*6 h (n = 6)*
Angiopoietin-like 4 (*Angptl4*)Rn.119611, NM_199115	20.38 *(p = 0.007)*	14.12 *(p = 0.005)*
Basic helix-loop-helix family, member (*Bhlhe40*) Rn.81055, NM_053328	No change	3.31 *(p = 0.000)*
Early growth response 1 (*Egr1*) Rn.9096, NM_012551	29.16 *(p = 0.006)*	6.67 *(p = 0.046)*
Immediate early response 3 (*Ier3*) Rn.23638, NM_212505	9.85 *(p = 0.001)*	No change
Plasminogen activator inhibitor type 1 (*Pai1*) Rn.29367, NM_012620	15.05 *(p = 0.023)*	No change
Solute carrier family 2 (facilitated glucose transporter), member 1 (*Slc2a1*, *Glut1*) Rn.3205, NM_138827	3.41 *(p = 0.002)*	No change

Only genes that showed at least a three-fold increase in expression with *p* < 0.05 were considered statistically significant

### Gene expression profiling of innate and adaptive immune response pathway genes

Because recent research has demonstrated cross-talk between hypoxia pathways and inflammatory pathways, we chose to also evaluate expression profiles for innate and adaptive immune response genes. At 3 h following *P. aeruginosa* LPS treatment, 11 genes showed a statistically significant up-regulation (*Cc112, Cc13, Cd14, Cxc110, Icam1, Il10, Il1b, Il6, Nfkbia, Tlr2,* and *Tnf)* and one gene was down-regulated (*Tlr5;* Table 2*).* Twelve genes were up-regulated at 6 h (*C3, Cc112, Cc13, Cd14, Il1b, Jak2, Nfkbia, Nlrp3, Slc11a1, Tlr1, Tlr2,* and *Tnf).* Seven genes remained elevated at both 3 and 6 h (*Cc112, Cc13, Cd14, Il1b, Nfkbia, Tlr2,* and *Tnf*).

**Table 2:** Changes in expression of genes involved in innate and adaptive immune responses after 3 and 6 h of LPS-induced inflammation

Gene Name	Fold Change		Gene Functions
	3 h	6 h	
Complement component 3 (*C3*) Rn.11378, NM_016994	No change	7.72 *(p = 0.002*)	Activation of the complement system.
Chemokine (C-C motif) ligand 12 (*Cc112)*; Rn.137780, NM_001105822	8.52 *(p = 0.000)*	26.15 *(p = 0.035*)	Angiogenesis; cellular response to fibroblast growth factor stimulus; participates in chemokine and cytokine mediated and cytokine signaling pathways.
Chemokine (C-C motif) ligand 3 (*Cc13*) Rn.10139, NM_013025	10.62 *(p = 0.000)*	14.63 *(p = 0.043*)	Monokine with inflammatory and chemokinetic properties.
CD 14 molecule *(Cd14)* Rn.42942, NM_021744	7.17 *(p = 0.002)*	6.67 *(p = 0.002)*	Accessory protein for TLR4.
Chemokine (C-X-C motif) ligand 10 (*Cxc110)*; Rn.10584, NM_139089	24.23 *(p = 0.000)*	NSS	Proinflammatory cytokine; may participate in T-cell effector function and development. Inhibits expression of StAR D1.
Intercellular adhesion molecule 1 (*1cam1*) Rn. 12, NM_012967	25.26 *(p = 0.000)*	NSS	Up-regulated by TLR2, TLR4, TLR5, and TLR6; ligand for leukocyte adhesion protein.
Interleukin 10 *(1110)* Rn.9868, NM_012854	4.43 *(p = 0.021)*	No change	Inhibits cytokine synthesis.
Interleukin 1 beta *(111b)* Rn.9869, NM_031512	11.32 *(p = 0.000)*	14.18 *(p = 0.053)*	Endogenous pyrogen, proinflammatory cytokine.
Interleukin 6 *(116)* Rn.9873, NM_012589	69.61 *(p = 0.000)*	NSS	Disrupts integrity of blood-testis-barrier by altering steady state levels of membrane proteins.
Janus kinase 2 (*Jak2*) Rn.18909, NM_031514	No change	3.77 *(p = 0.032)*	Non-receptor tyrosine kinase known to be involved in innate and adaptive immunity signaling.
Nuclear factor of kappa light polypeptide gene *(Nfkbia)* Rn.12550, NM_001105720	4.84 *(p = 0.000)*	3.99 (*p = 0.018*)	Inhibitor of NF-ĸB; binds NF-ĸB and retains it in the cytoplasm.
NLF family, pyrin domain containing 3 *(Nlrp3)* Rn.214177, XM_220513	No change	8.27 *(p = 0.001)*	No functional assignments in Uniprot.
Solute carrier family 11 (proton-coupled divalent; *Slc11a1)* Rn.105919, NM_001031658	No change	3.79 *(p = 0.000)*	Macrophage protein associated with resistance or susceptibility to intracellular pathogens.
Toll-like receptor 1 (*Tlr1)* Rn. 107,212, NM_001172120	No change	3.36 *(p = 0.018)*	Stimulated by bacterial lipoproteins.
Toll-like receptor 2 (*Tlr2)* Rn. 46,387, NM_198769	6.35 *(p = 0.000)*	8.70 (*p = 0.027*)	Increase expression of inflammatory cytokines IL-α, IL-6, and interferon-α, and β
Toll-like receptor 5 (*Tlr5)* Rn. 198,962, NM_001145828	−3.37 *(p = 0.052)*	No change	Increase expression of inflammatory cytokines IL-α, IL-6, and interferon-α, and β
Tumor necrosis factor (TNF superfamily, member 2; *Tnf*) Rn.2275,NM_012675	8.78 (*p* = 0.000)	4.58 (*p* = 0.029)	Chemokine-mediated signaling, induces germ cell apoptosis in autoimmune orchitis.

Genes were reported that showed more than a three-fold change following LPS-induced inflammation with *p* < 0.05. NSS, not statistically significant, greater than three-fold change observed with *p* > 0.05

### Pathway analysis of inflammatory genes affected by LPS-induced inflammation

DAVID Bioinformatics Resources 6.7 was utilized for functional annotation and pathway map analysis. This allowed for identification of multiple genes involved in specific signaling pathways. Of the inflammatory genes affected by LPS, 9 genes are part of the TLR signaling pathway (Additional file 1: Figure S1). With the exception of *Tlr5*, which was down-regulated 3 h following LPS-induced inflammation, genes circled in red were up-regulated at 3 and/or 6 h following LPS administration (Additional file 1: Figure S1).

## Discussion

This study was designed to begin to understand effects of *E. coli* and *P. aeruginosa* LPS-induced inflammation in activating HIF-1 and hypoxia pathway gene responses in the testis. Regulation of HIF-1 during inflammation has been studied in vivo and in vitro and reviewed extensively [23]. One hypothesis is that HIF-1 is up-regulated in microenvironments of inflamed tissues characterized by low levels of oxygen and glucose and high levels of inflammatory cytokines, reactive oxygen species (ROS), and nitrogen species, to protect cells against secondary inflammatory damage [32].

Steroidogenesis and spermatogenesis are inhibited by infection and inflammation of the testis [18]. For example, in mice using relatively high doses of LPS, Leydig cell steroidogenesis is inhibited via reduced synthesis of the cholesterol transport protein steroidogenic acute regulatory protein and steroidogenic enzymes [19]. To validate inflammation in our model system, serum testosterone levels were measured and decreases in serum testosterone after the 3 h and 6 h *P. aeruginosa* and 6 h *E. coli* LPS treatments confirmed LPS-induced inflammation and compromised testicular functions.

Previously we have shown that HIF-1 is highly expressed by normoxic Leydig cells and that, unlike other tissues, testicular HIF-1 expression is not induced by hypoxia [35]. This may be because physiologic oxygen tension of the testis is already hypoxic. Thus, studying the activation of a hypoxic response during inflammation in what may be an already hypoxic environment is of particular interest. In vitro studies have established the activation of hypoxia genes by LPS in multiple systems including pulmonary artery smooth muscle cells, macrophages, monocytes, and glioblastomas [33, 34, 36–38]. The increase in testicular HIF-1 protein we detected following LPS-induced inflammation indicates a role for HIF-1 and Leydig cells in the activation of (or protection against) an inflammatory response.

The accumulation of testicular HIF-1α protein following inflammation suggests crosstalk between inflammatory responses and hypoxic responses in the testis. Before determining the effects of HIF-1 up-regulation following LPS-induced inflammation, we chose to make an initial effort to identify potential pathways responsible for the crosstalk between inflammatory and hypoxic responses and up-regulation of HIF-1α. Studies suggest this crosstalk occurs via transcriptional regulation of HIF-1α by NF-κB, a key transcription factor responsible for mediating inflammatory responses [34, 37]. A functional NF-κB binding site in the HIF-1α promoter has been demonstrated [36] and LPS induces HIF-1 activation in human monocytes via NF-κB [33].

This led us to consider that NF-κB may be an important transcriptional activator of HIF-1α following LPS-induced inflammation in our model. EMSA experiments to measure NF-κB binding to the HIF-1α promoter revealed that binding activity decreased following LPS-induced inflammation. *Nfkb1* mRNA and protein expression did not increase and our pathway array results also demonstrated increased expression of *Nfkbia* mRNA*.* Taken together these results suggest that NF-κB is probably not a transcriptional activator of HIF-1α in the testis in vivo.

We also demonstrated that testicular HIF-1α is not up-regulated through an increase in *Hif-1α* mRNA. It is likely then that post-translational regulation of HIF-1α protein levels (via ubiquitination and protein stability) as occurs under hypoxic conditions, and not transcriptional regulation of HIF-1α is the likely mechanism for up-regulating HIF-1α protein levels in the testis during inflammation.

One of the functional clusters of inflammatory genes up-regulated in our study is the TLRs. In our work expression of *Tlr2* was shown to be elevated at both 3 and 6-h time points but *Tlr4* mRNA was not. Although others have demonstrated elevated expression of TLR-4 following LPS challenge of the testis [10]. But the absence of significant change in expression of *Tlr4* has been reported before. For example, Winnall et al. reported a significantly higher expression of TLR2 in relation to expression of the LPS receptor TLR4 and its co-receptor protein CD14 [7]. Sertoli cells have been shown to respond to bacteria through both TLR2 and TLR4 [39]. TLR4-dependent activation of HIF-1 in the lung has been demonstrated [40]. Our studies showed elevated expression of CD14. In addition, TLR stimulation elevates the synthesis of proinflammatory cytokines such as IL-1β and TNF (both of which showed elevated mRNA expression in our study) so perhaps TLR-dependent pathways and not NF-κB is the predominant activation pathway for HIF-1 in the testis. Recently we have also demonstrated that expression of a number of microRNAs in the testis are downregulated by LPS including miRNAs involved in regulating the NF-κB pathway [41].

Testicular macrophages are important for modulating inflammatory responses [1]. In testicular macrophages challenged with LPS, it has been shown that the NF-κB signaling pathway is blocked due to a lack of IκBα ubiquitination and degradation [42]. Rather LPS stimulates testicular macrophages to activate MAPK, AP-1, CREB and other pathways that increase production of proinflammatory cytokines such as IL-6 and TNF. Both IL-6 and TNF are known to have inhibitory roles on steroidogenesis of Leydig cells [1, 43] and so the elevated mRNA expression of these genes observed in our studies was expected. In this study up-regulated expression of mRNAs for proinflammatory cytokines such as IL-1 and IL-6, TNF underscores the importance of cytokine-mediated signaling pathways that are thought to be important for inflammatory responses of the testis, as demonstrated by other investigators [1].

Finally, the up-regulation of HIF-1α protein may also be caused by several physiologic changes. For example, a decrease in oxygen tension caused by inflammatory activity occurring in the testis may result in a hypoxic environment leading to HIF-1 activation. Similarly ROS released during inflammatory responses can also stabilize and elevate HIF-1α protein levels [23].

Results from hypoxia pathway arrays demonstrated that six hypoxic pathway genes (*Angptl4, Bhlhe40, Egr1, Ier3, Pai1,* and *Glut1)* are up-regulated 3 or 6 h following LPS-induced inflammation. Among 84 hypoxic genes, no genes appear to be down-regulated in response to LPS. Those that did show a significant increase do not appear to belong to any particular functional cluster. Due to the variety of functions exhibited by these genes, we do not want to speculate about the roles these genes may plan in inflammatory responses in the testis. These genes are the subject of future studies.

Although we did not determine the cell-type specific location of the genes analyzed in our study, in silico analysis of hypoxia pathways genes revealed that only *Bhlhe40* is known to be expressed in spermatogenic cells. *Angptl4, Egr1, Ier3,* and *Pai1* are expressed in myoid cells. *Pai1* is expressed in germ cells. *Egr1, Ier3, Pai1,* and *Slc2a1* are expressed in Sertoli cells, and *Egr1* is expressed in Leydig cells. These results suggest that supporting cells, and not germ cells, are primarily involved in inflammatory responses of the testis.

In addition, the literature includes few studies on hypoxic gene expression in the testis in response to LPS. Bourdon et al. detected *Pai1* expression in cultured Sertoli cells following LPS treatment [44]. Further experimental evidence is necessary to elucidate the specific mechanisms and functions of these genes, and will provide a greater understanding of why some appear to be early response genes while others may be late responders. These studies could also provide an explanation for why certain genes such as *Angptl4* and *Egr1* show a sustained response, with elevated expression at both 3 and 6-h time points.

While the up-regulation of HIF-1α suggests a role for the HIF-1 pathway in inflammatory responses of the testis, further studies are needed to determine possible HIF-1 target genes. HIF-1 was initially thought to play a significant role in the regulation of these hypoxic genes, but only *Glut1* is a known target gene for HIF-1. However, *Egr1, Ier3,* and *Pai1* are known target genes of NF-κβ. Also, although HIF-1 is known to activate over 100 target genes, not all targets were built into the array; therefore, we cannot exclude the possibility that there may be other HIF-1 target genes involved in inflammation.

## Conclusions

Regardless of the underlying reasons for HIF-1 activation, we conclude that the mechanism of HIF-1α protein accumulation following LPS-induced inflammation is not at the transcriptional level and likely not mediated through NF-κB. Further studies will be carried out to identify target genes regulated by HIF-1. This study provides a baseline analysis of hypoxia and inflammatory pathway gene expression changes following LPS-induced inflammation of the testis that will be a useful resource for researchers in the future. The range of genes affected and their potential functions, reflects the complexity of the inflammatory response of the testis and underscores that both hypoxic and inflammatory gene expression pathways are involved.

## Additional file

Additional file 1: **Figure S1.** Toll-like receptor signaling pathway including innate and adaptive immune response genes up-regulated or down-regulated by LPS-induced inflammation. (DOCX 359 kb)

## Abbreviations

ANOVA: analysis of variance
cAMP: cyclic AMP
cDNA: complementary DNA
C_t_: cycle threshold
DAVID: Database for Annotation, Visualization, and Integrated Discovery
DNA: deoxyribonucleic acid
EBNA: Epstein-Barr Nuclear Antigen
EBNA: Epstein-Barr Nuclear Antigen
EDTA: ethylene-Diamine-Tetra-Acetic acid
EMSA: Electrophoretic Mobility Shift Assays
HIF-1α: hypoxia-inducible factor-1 alpha
HRP: horseradish peroxidase
IL-1ß: interleukin beta
IκBα: inhibitory NFKB alpha
LPS: lipopolysaccharide
Mcl-1: myeloid leukemia cell differentiation 1
NFDM: nonfat dry milk
NIAID: National Institute of Allergy and Infectious Diseases
PBS: phosphate buffered saline
PCR: polymerase chain reaction
qPCR: real-Time Quantitative PCR
RNA: ribonucleic acid
ROS: reactive oxygen species
RT: reverse transcriptase
SDS-PAGE: sodium dodecyl sulfate polyacrylamide gel electrophoresis
TdT: terminal deoxynucleotidyl transferase
TLR: Toll-like receptor
TNF-α: tumor necrosis factor alpha
