# Supplementary material for: Effects of lipopolysaccharide-induced inflammation on hypoxia and inflammatory gene expression pathways of the rat testis
Source: Basic Clin Androl. 2018 Nov 16;28:14. doi: 10.1186/s12610-018-0079-x (PMC6238406; doi:10.1186/s12610-018-0079-x)
Supplement: Supplementary file 1 — Figure S1. Toll-like receptor signaling pathway including innate and adaptive immune response genes up-regulated or down-regulated by LPS-induced inflammation. (DOCX 359 kb) [file 12610_2018_79_MOESM1_ESM.docx]

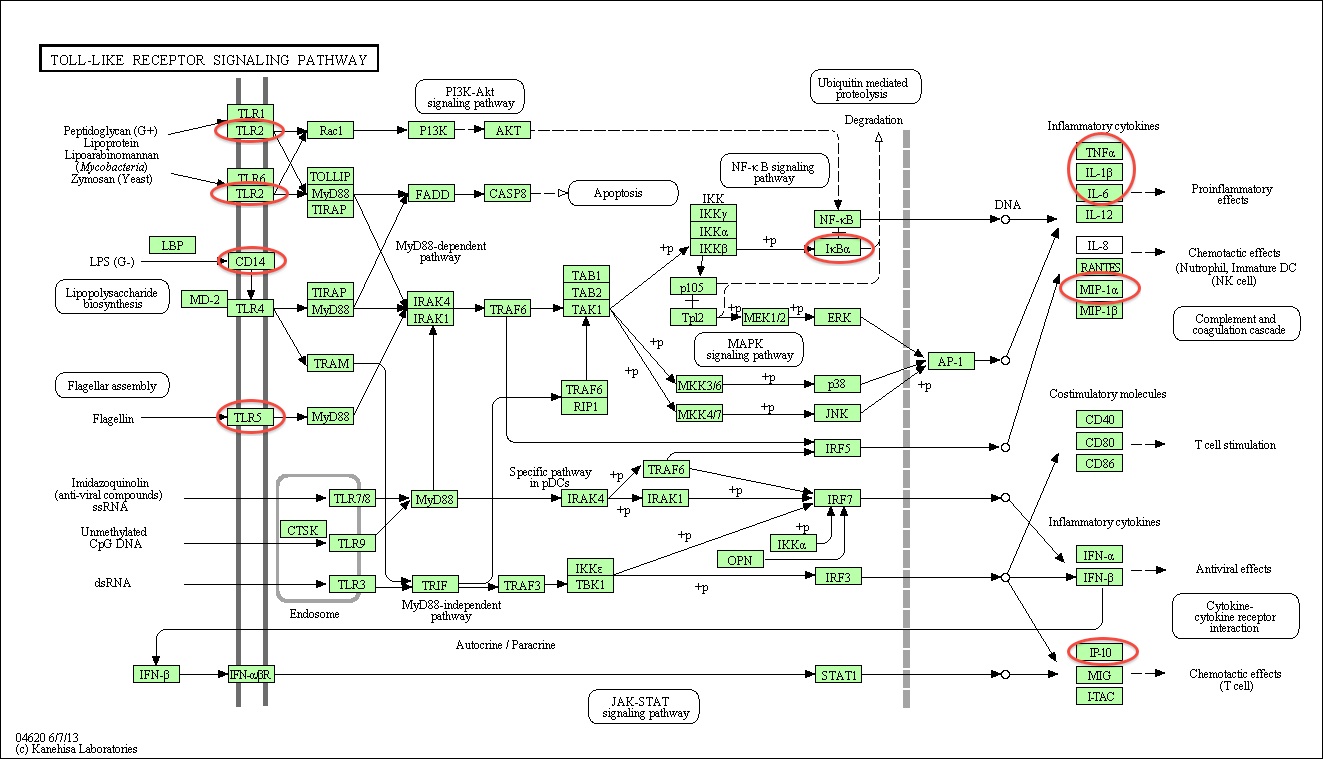


**Additional file 1: Figure S1: Toll-like receptor signaling pathway including innate and adaptive immune response genes up-regulated or down-regulated by LPS-induced inflammation.** The web-based DAVID Bioinformatics Resources 6.7 available through the National Institute of Allergy and Infectious Diseases (NIAID) was utilized for functional annotation and pathway map analysis. A functional clustering analysis was created based on the results of the RT-qPCR arrays. Overall, 9 genes from the innate and adaptive immune response pathway were clustered and are part of the toll-like receptor signaling pathway. With the exception of *Tlr5,* genes circled in red were up-regulated at 3 and/or 6 hours following LPS-induced inflammation.
